# Efficacy of GLP-1 Receptor agonists in treating Obstructive sleep apnea: A systematic review and meta-analysis of cardiometabolic and respiratory outcomes

**DOI:** 10.1007/s11325-026-03681-4

**Published:** 2026-04-24

**Authors:** Mrunalini Dandamudi, Larissa Lucena, Nicole Norma Gamarra-Valverde, Andrea Tripoli, Vinh Quang Tri Ho, Miguel Samaniego, Juan Pinilla, Macarena Lopez, Salomon Chamay, Juliana Giorgi, Alejandro Barbagelata, Edgardo Kaplinsky

**Affiliations:** 1 Montefiore Medical Center, Moses campus, New York Bronx, USA; 2 Federal University of Rio Grande do Norte, Natal, Brazil; 3 Universidad Peruana Cayetano Heredia, Faculdad de Medicina Alberto Hurtado, Lima, Peru; 4 Cardiac Intensive Care Unit, San Raffaele Hospital/Università Vita-Salute San Raffaele, Milan, Italy; 5 Faculty of Medicine, University of Debrecen, Debrecen, Hungary; 6 Universidad Autónoma Metropolitana, Ciudad de México, Mexico; 7 CES University, School of Medicine, Medellín, Colombia; 8 Universidad Catolica de Buenos Aires, Buenos Aires, Argentina; 9 Morristown Medical center, NJ Morristown, USA; 10 Hospital Sirio Libanes, São Paulo, São Paulo, Brazil; 11 Albert Einstein Hospital, São Paulo, São Paulo, Brazil; 12 Duke University, North Carolina Durham, USA; 13 Hospital Municipal de Badalona, Barcelona, Spain; 14 Fellow, Advanced Heart Failure and Transplant Cardiology Montefiore Einstein medical centre, New York Bronx, USA

**Keywords:** Obstructive sleep apnea, GLP-1 receptor agonists, Weight loss, Apnea-hypopnea index, Metabolic effects, Body mass index, Meta-analysis

## Abstract

**Background:**

Obstructive sleep apnea (OSA) is strongly associated with obesity, and weight loss is a cornerstone of its management. Glucagon-like peptide-1 receptor agonists (GLP-1 RAs), are a class of medications that induce significant weight loss and have shown promise in improving OSA severity.

**Purpose:**

This meta-analysis aimed to evaluate the pooled efficacy of GLP-1 RAs on sleep-disordered breathing, body weight, and cardiovascular risk factors in patients with OSA.

**Methods:**

A systematic review and meta-analysis of four randomized controlled trials were performed, comparing GLP-1 RA therapy with placebo in adults with OSA. The primary outcomes analyzed were the mean differences in the change from baseline for the apnea-hypopnea index (AHI), body weight, and systolic and diastolic blood pressure.

**Results:**

In the pooled analysis, treatment with GLP-1 RAs was associated with a robust and statistically significant reduction in AHI (mean difference [MD]: -13.89 events/hour; *p* < 0.01). Therapy also resulted in substantial weight loss compared to placebo (MD: -12.46 kg; *p* < 0.01). For cardiovascular parameters, the use of GLP-1 RA was associated with a significant reduction in systolic blood pressure (mean difference [MD]: -4.86 mmHg; *p* < 0.01) and diastolic blood pressure (*p* = 0.03).

**Conclusion:**

GLP-1 receptor agonists significantly reduce OSA severity, promote substantial weight loss, and lower systolic and diastolic blood pressure in patients with obstructive sleep apnea and obesity. These findings support the role of GLP-1 RAs as a multifaceted and effective therapeutic intervention for managing OSA.

**Graphical abstract:**

**Figure:**
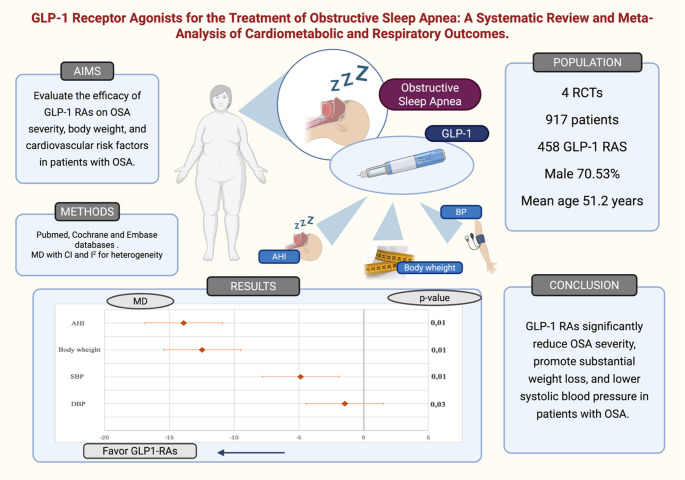
GLP-1: Glucagon-like peptide-1; OSA: Obstructive sleep apnea; MD: Mean difference; CI: confidence interval; RCTs: randomized controlled trials, GLP-1 RAS: Glucagon-like peptide-1 receptor agonists; AHI: apnea-hypopnea index; SBP: systolic blood pressure, DBP: diastolic blood pressure. Illustration created with BioRender

## Introduction

Obstructive sleep apnea (OSA) is a prevalent and often underdiagnosed chronic disorder characterized by repetitive episodes of pharyngeal collapse during sleep. These episodes result in recurrent apneas and hypopneas, leading to intermittent hypoxemia, hypercapnia, and frequent arousals from sleep [1]. The severity of OSA is quantified by the apnea-hypopnea index (AHI), which measures the number of respiratory events per hour of sleep. An AHI of 15 to 29.9 events per hour is classified as moderate OSA, while an AHI of 30 or more events per hour indicates severe disease [2]. This condition is not merely a sleep disturbance; it is a significant independent risk factor for major cardiovascular complications, including hypertension and cardiovascular mortality, and is associated with reduced quality of life and an increased risk of accidents [2].

The pathophysiology of OSA is deeply intertwined with obesity, with excess adiposity considered the most critical reversible risk factor [1, 2]. The relationship appears to be bidirectional, where obesity promotes the development and progression of OSA; the sleep fragmentation and hypoxia associated with OSA can, in turn, exacerbate metabolic dysfunction [2]. It is estimated that approximately 60–70% of individuals with OSA are overweight, and a substantial portion of moderate to severe OSA cases are directly attributable to excess weight [2]. Consequently, clinical practice guidelines consistently recommend weight management as a foundational therapeutic strategy for patients with OSA and obesity [1–3]. Although excess adiposity is a critical reversible risk factor, OSA pathophysiology is multifactorial, encompassing other elements such as craniofacial anatomy, upper airway neuromuscular control, and central respiratory drive. Recognizing these diverse mechanisms emphasizes that OSA is not solely a disease of obesity and underscores why therapeutic responses may vary according to the dominant underlying physiological endotypes [4–6]. This broader framework sets the stage for a more comprehensive understanding of how weight-modifying therapies such as GLP-1 receptor agonists may interact with other determinants of OSA severity.

In the pursuit of effective weight management strategies, glucagon-like peptide-1 (GLP-1) receptor agonists have emerged as a powerful pharmacological tool for achieving this goal. These agents, which include liraglutide and the dual gastric inhibitory peptide (GIP)/GLP-1 receptor agonist tirzepatide, induce weight loss primarily by targeting the central nervous system to reduce appetite and increase satiety [2, 7]. Their mechanism involves modulating key neural pathways that regulate hunger, as well as a peripheral action that delays gastric emptying, contributing to a feeling of fullness and a subsequent reduction in overall energy intake [2, 7].

The established link between weight loss and the amelioration of OSA provides a strong rationale for investigating GLP-1 receptor agonists (GLP-1 ARs) as a treatment for this condition. Studies have consistently demonstrated that weight loss, whether achieved through lifestyle interventions, pharmacotherapy, or bariatric surgery, significantly reduces OSA severity [2]. This improvement is characterized by a clinically significant reduction in the AHI, increased blood oxygen saturation, and improvements in patient-reported outcomes related to sleep quality and daytime function [1]. Given that the therapeutic effect of weight loss on OSA is often proportional to the amount of weight lost, the substantial weight reduction observed with GLP-1 RAs positions them as a promising, non-invasive treatment modality that could fundamentally alter the management of OSA in patients with obesity [1, 2].

Building on this rationale, our meta-analysis aims to systematically evaluate the impact of GLP-1 RAs on the severity of OSA, with a focus on changes in AHI and related clinical outcomes. By synthesizing available evidence, we seek to clarify the potential of these agents not only as metabolic interventions but also as targeted therapy for OSA in individuals with obesity. Given the growing burden of OSA and its systemic consequences, alongside the rising clinical adoption of GLP-1 therapies for weight loss and cardiometabolic protection, this analysis addresses a timely and clinically relevant question. The findings have the potential to inform guideline updates and enhance therapeutic strategies, bridging the fields of sleep medicine, endocrinology, and cardiometabolic care.

## Methods

This meta-analysis followed the Cochrane Collaboration Handbook for Systematic Reviews of Interventions and the Preferred Reporting Items for Systematic Reviews and Meta-analysis (PRISMA) statement guideline. The prospective meta-analysis protocol was registered at the International Prospective Register of Systematic Reviews (PROSPERO; registration 1089356).

### Data source and search strategy

We systematically searched the PubMed/MEDLINE, EMBASE, and Cochrane databases for published studies from their inception to June 25, 2025. Two authors (L.L. and M.D.) conducted the systematic review of results independently, and disagreements were resolved in a discussion between the authors.

### Eligibility criteria

Studies were considered eligible if: 1) they were randomized controlled studies (RCTs); 2) compared GLP-1 agonists versus placebo or standard of care treatment; 3) included patients with OSA; and 4) reported data for at least one of the prespecified efficacy and safety endpoints of interest. We excluded studies that 1) were single-arm studies, abstracts, case series, and case reports; 2) did not report any outcome of interest; 3) had overlapping populations; and 4) were non-English language studies.

### Outcomes

The primary efficacy endpoint was AHI. Secondary outcomes included changes in body weight and changes in diastolic and systolic blood pressure. Subgroup analyses were made based on different classes of drugs and continuous positive air pressure (CPAP) use. The pooled analysis of safety outcomes, specifically adverse events and mortality, was not possible due to the lack of harmonized and consistently reported safety data across the included studies. While individual trials, such as Blackman et al., Malhotra et al., and Jiang et al., reported various safety endpoints, including adverse events, serious adverse events, and mortality, these outcomes were not uniformly defined or presented in a manner that allowed for quantitative synthesis. This limitation precluded the ability to draw robust, comparative conclusions regarding the safety profile of GLP-1–based therapies in this population.

### Quality assessment

RCT quality assessment was performed using the Cochrane Risk of Bias 2 (RoB-2) tool for randomized controlled trials (RCTs). Two authors (J.P. and M.D.) independently conducted the risk of bias assessment, and any disagreements were resolved through consensus.

### Statistical analysis

We summarized binary endpoints using the Mantel-Haenszel random-effects model, with a 95% confidence interval (CI) as a measure of effect size. The DerSimonian-Laird method was used to calculate the between-study variance. Heterogeneity was assessed using Cochrane’s Q statistic and I^2^ statistics. We determined the consistency of the studies based on I^2^ values of 0%, ≤ 25%, ≤ 50%, and > 50%, indicating no heterogeneity, low heterogeneity, moderate heterogeneity, and substantial heterogeneity, respectively. All tests were two-tailed, and a p-value of < 0.05 was considered statistically significant. We used R version 4.4.1 (R Foundation for Statistical Computing, Vienna, Austria) and the extension package “meta” for all calculations and graphics.

## Results

### Study selection and baseline characteristics

**Fig. 1:**
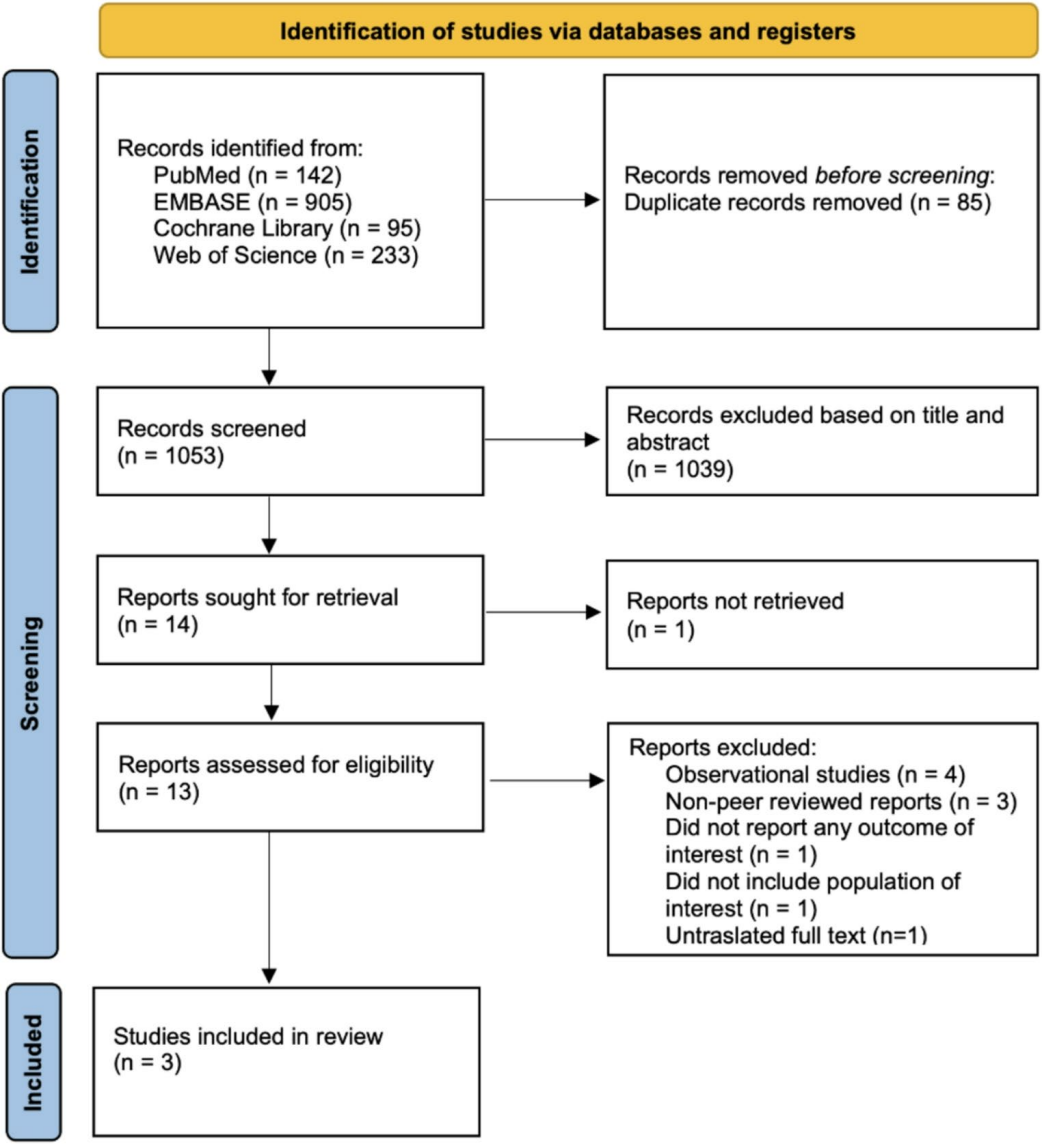
PRISMA Flowchart

The initial search yielded 1375 results. After removing duplicates and unrelated studies based on title and abstract, we retrieved 13 studies for full-text review (Fig. 1). Of those, 4 RCTs [1, 2, 4] as SURMONT-OSA Trial by Malhotra et al. [2] comprehends 2 RCTs in a single publication without overlapping population, met the inclusion criteria, involving a total of 917 patients: 458 in the GLP-1 receptor agonist group and 459 in the placebo or standard treatment group, being 70.53% males and mean age 51.2 years. The baseline characteristics of the studies are outlined in Tables 1 and 2.

**Table 1:** Characteristics of included studies

Study	Total Number of Patients	GLP-1 (*n*)	Placebo (*n*)	Type of GLP-1	Inclusion Criteria	Highest Intervention Dose, Injection Route	Follow-up Duration	Primary Outcomes
Blackman 2016	359	180	179	Liraglutide	Patients aged 18–64 years with a stable body weight and body mass index (BMI) of ≥ 30 kg/m^2^. Eligible individuals had to be diagnosed with moderate or severe OSA and be unable or unwilling to use CPAP therapy.	3.0 mg Subcutaneous	8 months	Liraglutide was generally well accepted as an adjunct to diet and exercise, and in persons with obesity and moderate to severe OSA, it significantly reduced AHI, body weight, SBP, and HbA1c compared to a placebo.
Jiang 2022	89	44	45	Liraglutide	Patients diagnosed with T2DM with moderate to severe OSA of AHI ≥ 15. No current use of DPP-4 inhibitors or GLP-1 treatment. Current use of CPAP treatment.	1.8 mg Subcutaneous	3 months	In T2DM patients with severe OSA, liraglutide plus CPAP can successfully lower mean systolic blood pressure, lower BMI, and improve hypoxia and AHI scores.
Molharta 2024	469	234	235	Tirzepatide	Patients diagnosed with moderate to severe OSA (AHI ≥ 15) and obesity (BMI ≥ 30). Have a history of at least 1 self-reported unsuccessful dietary effort to lose body weight.	10 mg or 15 mg Subcutaneous	12 months	Tirzepatide enhanced sleep-related patient-reported outcomes and decreased body weight, hypoxic burden, systolic blood pressure, hsCRP concentration, and AHI in individuals with moderate to severe OSA and obesity.

Abbreviations** =** GLP-1: Glucagon-Like Peptide-1; BMI: Body Mass Index; OSA: Obstructive Sleep Apnea; AHI: Apnea-Hypopnea Index; CPAP: Continous Positive Airway Pressure; T2DM: Type 2 Diabetes Mellitus; hsCRP: high-sensitivity C-Reactive Protein; mg: milligram; kg: kilogram; SBP: Systolic Blood Pressure: HbA1c: Glycosylated Hemoglobin; DDP-4: Dipeptidyl Peptidase-4

**Table 2:** Baseline characteristics of patients across all the studies

Study	Age ^†^	Country	Male, n (%)	BMI kg/m^2†^	Hypertension, n (%)	AHI, events/h^†^	OSA Severity, n (%)	Dyslipidemia, n (%)	HbA1c, n (%)^†^	SBP, mmHg^†^	DBP, mmHg^†^
Blackman 2016	48.6 ± 9.9	Canada	129 (71.8)	39.2 ± 6.9	152 (42.3)	49.2 ± 27.5	Moderate (AHI ≥ 15): 118 (32.9) Severe (AHI ≥ 30): 241 (67.1)	120 (33.4)	5.7 ± 0.7	126.5 ± 11.9	81.7 ± 8.2
Jiang 2022	55.2 ± 6.5	China	63 (70.1)	26.8 ± 3.5	67 (75.3)	30.5 ± 6.8	Moderate to Severe (AHI ≥ 15): 89 (100)	N/A	6.6 ± 0.6	131.3 ± 12.4	73.7 ± 8.5
Molharta 2024	49.8 ± 11.4	USA	327 (68.7)	38.9 ± 6.5	359 (76.5)	50.5 ± 28.9	Moderate (AHI ≥ 15): 154 (32.8) Severe (AHI ≥ 30): 306 (65.2)	386 (82.3)	5.6 ± 0.4	130.0 ± 12.5	82.8 ± 8.7

† = Median or Mean

Abbreviations: BMI: Body Mass Index; AHI: Apnea-Hypopnea Index; OSA: Obstructive Sleep Apnea; SBP: Systolic Blood Pressure; DBP: Diastolic Blood Pressure; mmHg: millimeters of mercury; HbA1c: Glycosylated Hemoglobin; USA: United States of America; N/A: Not APplicable; kg: kilogram.Abbreviations: BMI: Body Mass Index; AHI: Apnea-Hypopnea Index; OSA: Obstructive Sleep Apnea; SBP: Systolic Blood Pressure; DBP: Diastolic Blood Pressure; mmHg: millimeters of mercury; HbA1c: Glycosylated Hemoglobin; USA: United States of America; N/A: Not APplicable; kg: kilogram

### Primary endpoints

**Fig. 2:**
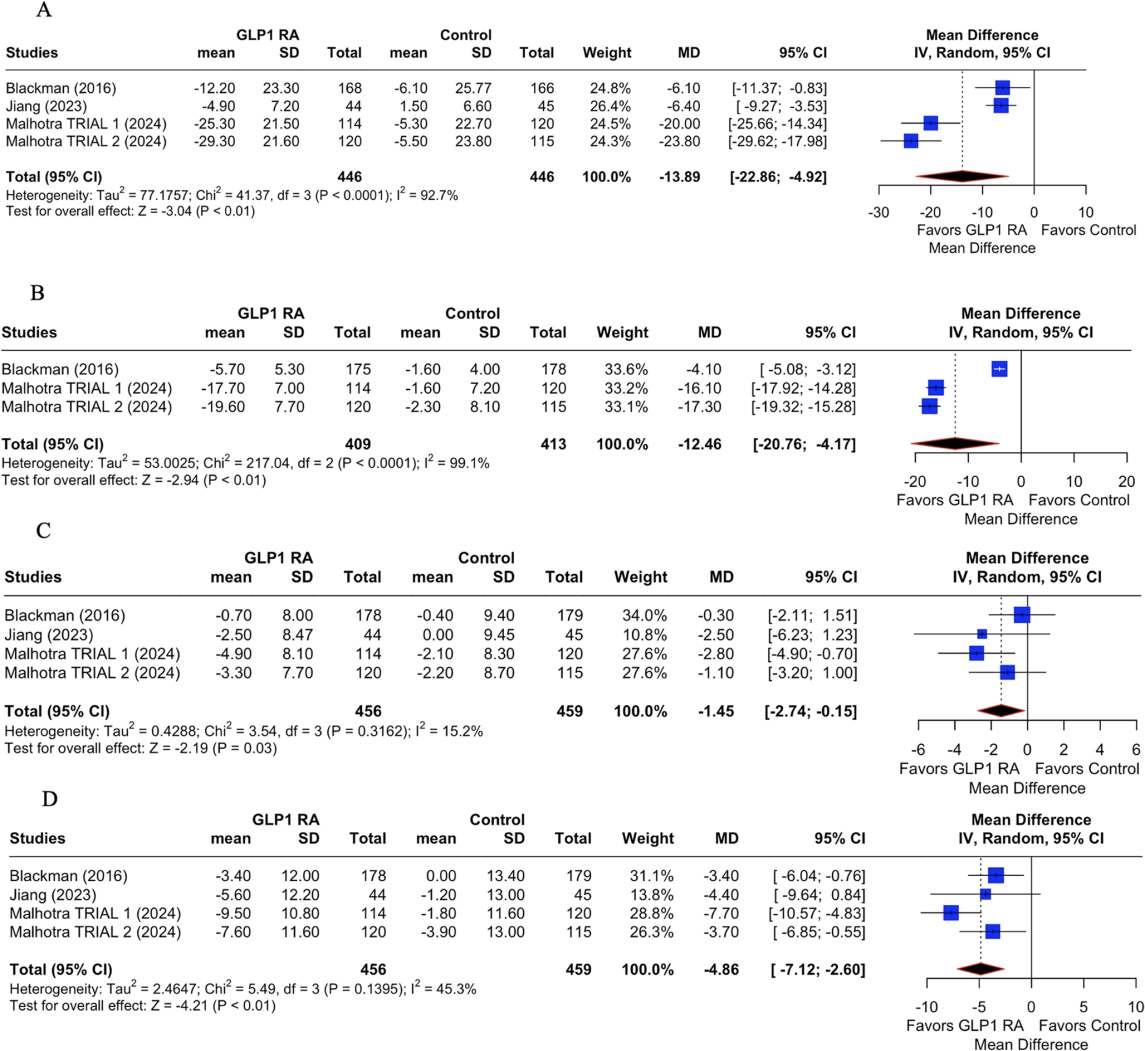
Primary and secondary outcomes of GLP-1 on sleep apnea. (**A**): Apnea Hypopnea Index (AHI); (**B**) Change in body weight; (**C**) Change in Diastolic blood pressure; (**D**) Change in Systolic blood pressure. GLP-1 RA: GLP-1 receptor agonist; MD: mean difference; CI: confidence interval

In the pooled analysis evaluating the impact of GLP-1 RAs on patients with OSA, significant clinical benefits were observed. The primary outcome, AHI, showed a robust and statistically significant reduction with GLP-1 therapy (Fig. 2.A), with a mean difference (MD) of −13.89 events/hour (95% CI: −22.86 to −4.92; *p* < 0.01, I^2^= 92.7%), indicating a meaningful improvement in OSA severity.

### Secondary endpoints

Secondary outcomes also demonstrated favorable effects of GLP-1 RAs. There was a substantial reduction in body weight (Fig. 2.B), with a pooled MD of −12.46 kg (95% CI: −20.76 to −4.17; *p* < 0.01; I^2^ = 99.1%), which likely contributed to the observed improvements in respiratory status. Regarding cardiovascular parameters, GLP-1 treatment was associated with a significant reduction in systolic blood pressure (Fig. 2.D) of −4.86 mmHg (95% CI: −7.12 to −2.60; *p* < 0.01; I^2^= 45.3%), such as change in diastolic blood pressure (MD: −1.45 mmHg; 95% CI: −2.74 to −0.15; *p* = 0.03; I^2^ = 15.2%; Fig. 2.C).

Collectively, these findings support the use of GLP-1 RAs as a multifaceted therapeutic strategy in OSA management, offering concurrent benefits in sleep-disordered breathing, weight reduction, and blood pressure control.

### Subgroup analysis

#### Between drug classes

**Fig. 3:**
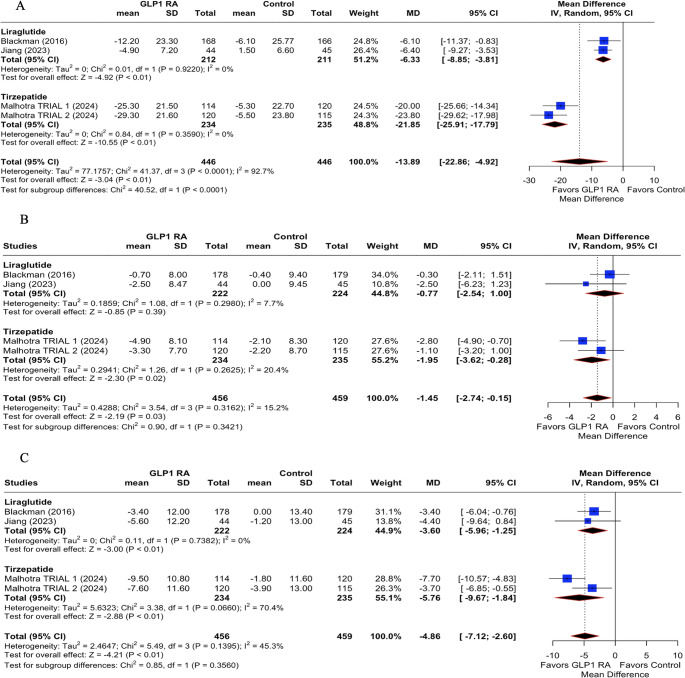
Subgroup analysis of GLP-1 on sleep apnea based on different classes of drugs. (**A**): Apnea-Hypopnea Index (AHI); (**B**) Change in Diastolic blood pressure; (**C**) Change in Systolic blood pressure. GLP1RA: GLP-1 receptor agonists; MD: mean difference; CI: confidence interval

Subgroup analysis of the change in AHI revealed significant differences between drug classes (Fig. 3.A). Liraglutide-based interventions [2, 4] showed a moderate but statistically significant reduction in AHI (MD: −5.20 events/hour; 95% CI: −7.89 to −2.51; *p* < 0.01; Fig. 3.A). In contrast, tirzepatide, a dual GLP-1 RA (GIP/GLP-1 RA) [1], was associated with a markedly larger effect, with a pooled mean difference of **-**23.80 events/hour (95% CI: −29.62 to −17.98; *p* < 0.01). The test for subgroup differences was statistically significant (*p* < 0.0001), indicating a more substantial reduction in AHI with tirzepatide compared to liraglutide.

Regarding diastolic blood pressure, liraglutide did not produce a significant change compared to placebo (MD: −0.77 mmHg; 95% CI: −2.54 to 1.00; *p* = 0.39; Fig. 3.B). In contrast, tirzepatide showed a statistically significant reduction (MD: −1.95 mmHg; 95% CI: −3.62 to −0.28; *p* = 0.02). However, the test for subgroup differences was not significant (*p* = 0.34), suggesting that while tirzepatide showed a greater numerical benefit, the difference between the drugs was not statistically robust, likely due to the dual agonist mechanism.

For systolic blood pressure, liraglutide therapy led to a moderate and significant reduction (MD: **-**4.00 mmHg; 95% CI: −6.25 to −1.75; *p* < 0.01; Fig. 3.C), whereas tirzepatide again showed a more pronounced effect (MD: −5.76 mmHg; 95% CI: −7.97 to −3.54; *p* < 0.01). Despite this apparent difference in magnitude, the test for subgroup differences was not statistically significant (*p* = 0.36), indicating that both agents may effectively reduce systolic blood pressure, without a superior drug.

### Stratified by CPAP use

**Fig. 4:**
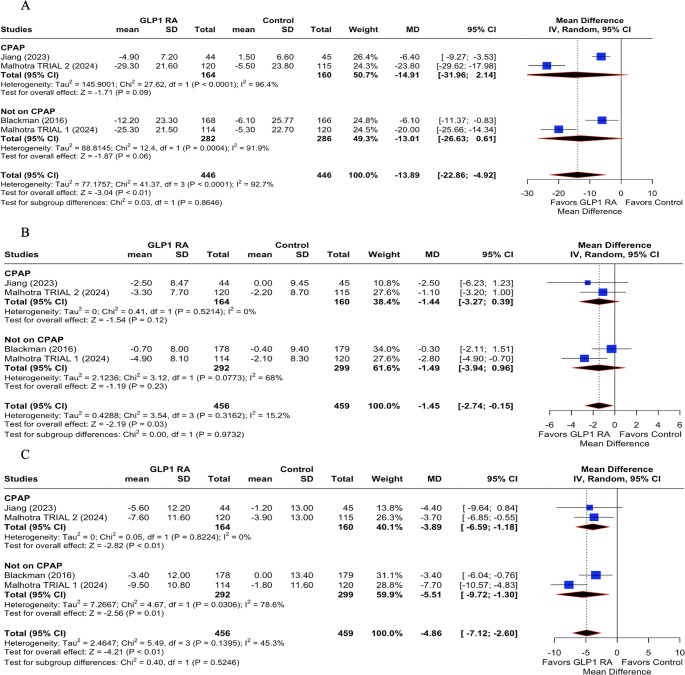
Subgroup analysis of GLP-1 on sleep apnea based on continuous positive airway pressure (CPAP) use. (**A**): Apnea-Hypopnea Index (AHI); (**B**) Change in Diastolic blood pressure; (**C**) Change in Systolic blood pressure. GLP1RA: GLP-1 receptor agonists; MD: mean difference; CI: confidence interval

Subgroup analysis stratified by CPAP use revealed that GLP-1 RAs significantly reduced AHI regardless of whether participants were on CPAP therapy. In patients using CPAP [1, 4], the pooled mean difference was − 14.91 events/hour (95% CI: −31.96 to 2.14; *p* = 0.09; Fig. 4A), which did not reach statistical significance, possibly due to wider confidence intervals and heterogeneity (*I²* = 94.6%). In contrast, in patients not receiving CPAP [1, 2], GLP-1 therapy produced a significant reduction in AHI (MD: −13.01 events/hour; 95% CI: −20.63 to −5.39; *p* < 0.01; Fig. 4.A). However, the test for subgroup differences was not significant (*p* = 0.86), suggesting no clear interaction between CPAP use and the effect of GLP-1 RAs on AHI.

In patients on CPAP, the pooled effect of GLP-1 RAs on diastolic blood pressure was not statistically significant (MD: −1.44 mmHg; 95% CI: −3.27 to 0.39; *p* = 0.12; Fig. 4.B). Similarly, among those not using CPAP, GLP-1 RAs showed a trend toward significance (MD: −1.49 mmHg; 95% CI: −2.94 to −0.05; *p* = 0.06, Fig. 4.B). Nevertheless, the test for subgroup differences was not significant (*p* = 0.97), indicating that CPAP status did not significantly modify the effect of GLP-1 therapy on diastolic pressure.

For systolic blood pressure, GLP-1 RA treatment was associated with a significant reduction in both subgroups. Among patients using CPAP, the mean difference was − 5.61 mmHg (95% CI: −9.45 to −1.78; *p* < 0.01; Fig. 4.C), while in those not on CPAP, the reduction was − 4.26 mmHg (95% CI: −6.39 to −2.14; *p* < 0.01; Fig. 4.C). The test for subgroup differences again showed no significant interaction (*p* = 0.52), indicating a consistent blood pressure–lowering effect of GLP-1 RAs, regardless of CPAP usage.

### Sensitivity analysis

**Fig. 5:**
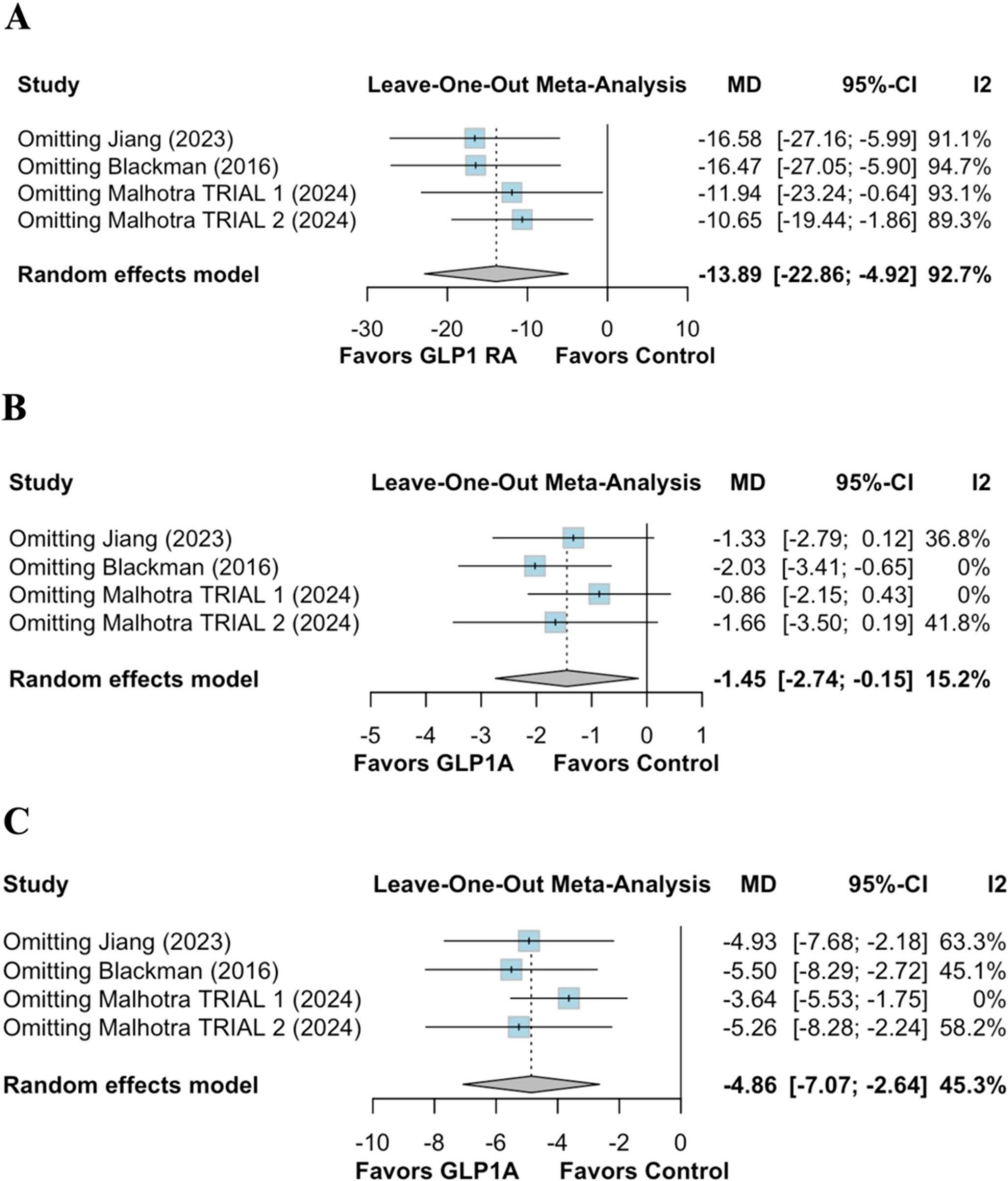
Sensitivity analysis through leave-one-out. (**A**) Apnea-hypopnea index (AHI); (**B**) Outcome of diastolic blood pressure; (**C**): Outcome of systolic blood pressure. GLP-1RA: GLP-1 receptor agonists; MD: mean difference; CI: confidence interval

To assess the robustness of our findings, we conducted leave-one-out sensitivity analyses for the primary and secondary outcomes. The primary outcome, change in AHI, remained statistically significant across all iterations, with pooled MD ranging from − 10.65 to −16.58 events/hour (Fig. 5.A). The overall effect size (MD: −13.89; 95% CI: −22.86 to −4.92) was consistent, indicating that no single study disproportionately influenced the result. Similarly, for the secondary outcome of diastolic blood pressure (Fig. 5.B), the pooled estimate remained directionally stable. However, statistical significance was sensitive to the exclusion of individual trials, especially Blackman et al. [2], where its omission slightly attenuated the effect (MD: −2.03 mmHg; 95% CI: −3.41 to −0.65). In contrast, the impact of GLP-1 RAs on systolic blood pressure (Fig. 5.C) was consistently significant across all leave-one-out analyses, with MDs ranging from − 3.64 to −5.50 mmHg, further affirming the robustness of the blood pressure–lowering effect.

Collectively, these sensitivity analyses support the stability and reliability of the meta-analytic findings, particularly for AHI and systolic blood pressure outcomes, reinforcing the potential of GLP-1 RAs as a therapeutic intervention in OSA.

### Quality assessment

The risk of bias assessment for RCTs using the RoB-2 tool in this review reveals an overall low risk of bias across studies evaluating GLP-1 RAs for the treatment of OSA (Table 3). Most studies demonstrated a low risk in key domains, such as the randomization process and missing outcome data, indicating a robust trial design and adequate follow-up. However, some concerns were noted in the domain of deviations from intended interventions, particularly in open-label studies where blinding of participants and personnel was not feasible, which could potentially introduce performance bias. Additionally, a few studies presented some concerns regarding the measurement of the outcome due to the subjective nature of specific assessments or the lack of assessor blinding. Nevertheless, outcome reporting was generally consistent and transparent across trials, minimizing the risk of selective reporting. Overall, the included RCTs were judged to be of acceptable methodological quality, supporting the validity of the pooled findings in this meta-analysis.

**Table 3:** Risk of bias summary for randomized studies (RoB-2)

Study	Bias from randomization process	Bias due to deviations from intended interventions	Bias due to missing outcome data	Bias in measurement of the outcomes	Bias in selection of the reported result	Overall risk of bias
Jiang 2023	Low	Low	Low	Low	Low	Low
Blackman 2016	Low	Low	Low	Low	Low	Low
Malhotra trial 1	Low	Low	Low	Low	Low	Low
Malhotra trial 2	Low	Low	Low	Low	Low	Low

## Discussion

This meta-analysis shows that GLP-1 RAs offer significant clinical benefits for patients with OSA. GLP-1 RAs resulted in a substantial reduction in AHI (*p* < 0.01), accompanied by significant reduction in body weight (–12.46 kg), systolic blood pressure (–4.86 mmHg) and diastolic pressure showed a modest, but statistically significant reduction (–1.45 mmHg; *p* = 0.03). Subgroup analysis revealed that tirzepatide produced a greater reduction in AHI than liraglutide (*p* < 0.0001), with comparable effects on blood pressure. These benefits were consistent regardless of CPAP use, supporting GLP-1 RAs as a promising adjunct in the management of OSA.

OSA is characterized by recurrent upper airway collapse during sleep, leading to cyclical hypoxemia, hypercapnia, and sleep fragmentation. Globally, nearly 1 billion adults (age 30–69) experience mild to severe OSA, including approximately 425 million with moderate-to-severe disease (AHI ≥ 1) [8]. In the U.S., moderate to severe OSA affects approximately 15–30% of adults. Obesity affects nearly 2 billion adults worldwide and over 45% in the U.S. An estimated 60–70% of OSA patients are obese, and as many as 78% of bariatric surgery candidates have OSA [9]. Given this close association, a substantial number of obese individuals likely suffer from undiagnosed OSA, underscoring the need for proactive screening in obesity care.

Obesity-driven OSA contributes significantly to healthcare expenditure due to increased hospitalizations, comorbid disease management, and long-term complications. In the U.S. alone, obesity-related conditions, including OSA, account for ~$172 billion annually [10]. Globally, direct and indirect costs linked to obesity and related sleep disorders reach into the hundreds of billions, reflecting the burden from cardiovascular disease, diabetes, and medical utilization. Addressing obesity through effective interventions could substantially reduce this financial strain on healthcare systems [10].

Obesity is the major risk factor for OSA, primarily due to the deposition of adipose tissue in the pharyngeal walls, tongue, and abdominal cavity, which increases upper airway collapsibility and reduces lung volume during sleep, predisposing individuals to airway obstruction [11, 12]. Conversely, OSA itself contributes to weight gain and metabolic dysregulation, establishing a self-perpetuating cycle. The intermittent hypoxia and sleep fragmentation characteristic of OSA activate the sympathetic nervous system and alter hormonal pathways, resulting in increased ghrelin and decreased leptin levels, which promote appetite and reduce energy expenditure [13, 14]. This leads to progressive weight gain, further exacerbating the severity of OSA and reinforcing the pathophysiologic loop. Additionally, OSA-induced oxidative stress, systemic inflammation, and endothelial dysfunction promote insulin resistance, contributing to the development of metabolic syndrome and atherosclerosis [15]. These changes increase arrhythmic susceptibility, particularly atrial fibrillation, and accelerate the progression of heart failure [16–19]. Therefore, OSA and obesity are deeply interconnected in a bidirectional and reinforcing manner, collectively elevating the risk of cardiometabolic morbidity and mortality [11].

GLP-1 RAs, such as liraglutide and semaglutide, are well-established agents for promoting weight loss through multiple mechanisms, including enhanced satiety, delayed gastric emptying, reduced appetite, and glucose-dependent insulin secretion [20]. More recently, dual GLP-1/glucose-dependent insulinotropic polypeptide (GIP) receptor agonists, like tirzepatide, have emerged as a novel class that combines the actions of both incretin hormones. By activating the GIP receptor in addition to GLP-1 pathways, these agents amplify insulin secretion, modulate energy balance, and significantly enhance weight reduction outcomes [21]. Clinical trials, including the SURPASS program, have shown that tirzepatide achieves greater reductions in body weight (up to 22.5%) and HbA1c levels compared to GLP-1 monotherapy with semaglutide [22, 23]. The synergistic effects of dual agonism offer a promising therapeutic strategy for obesity and type 2 diabetes (T2DM) management.

GLP-1 RAs have emerged as promising agents in the treatment of OSA, primarily through their effects on weight reduction and metabolic modulation. Blackman et al. [2] demonstrated that liraglutide 3.0 mg significantly reduced the AHI by 12.2 events/hour and induced an average weight loss of 5.7% over 32 weeks in obese patients with moderate to severe OSA, even in the absence of CPAP therapy [2]. This study established the proof of concept that GLP-1 RAs can improve OSA severity through mechanisms that extend beyond glucose control. Further evidence, as reported by Jiang et al., confirmed that liraglutide treatment led to significant reductions in AHI and improvements in glycemic markers and inflammatory profiles in patients with T2DM and severe OSA [7]. These findings support the hypothesis that GLP-1 therapy, by reducing central adiposity, modulating sympathetic activation, and decreasing systemic inflammation, can ameliorate the underlying pathophysiology of OSA [2].

Recent advances have further highlighted the superior efficacy of dual GLP-1/GIP receptor agonists in managing OSA. The SURMOUNT-OSA phase 3 trial by Malhotra et al. [1] demonstrated that tirzepatide (GLP-1/GIP RA) resulted in a significant reduction in AHI (up to 29.3 events/hour) and achieved an average weight loss of 19.6% over 52 weeks [1]. Additionally, tirzepatide significantly improved hypoxic burden, lowered C-reactive protein (CRP), and reduced systolic blood pressure, underscoring its broad cardiometabolic benefits. Collectively, these high-impact studies emphasize the role of incretin-based therapies in treating OSA by targeting its metabolic drivers. Dual agonists like tirzepatide, previously approved for obesity and type 2 diabetes, recently received FDA approval for the treatment of moderate-to-severe OSA in adults with obesity. This landmark approval, based on the robust results of the phase 3 SURMOUNT-OSA trials, marks the first pharmacologic therapy specifically approved for OSA in this population. It reflects a significant advance in the management of OSA beyond conventional CPAP therapy, particularly in patients where weight and metabolic factors play a central role. The approval underscores the growing recognition of GLP-1–based therapies as effective agents not only in metabolic regulation but also in improving sleep-disordered breathing outcomes in obesity-related OSA [24].

The benefits of these pharmacotherapies extend beyond clinical metrics to meaningful improvements in patients’ daily lives. OSA is associated with significant daytime hypersomnolence, reduced quality of life, and impaired functioning [2]. Treatment with these agents has been shown to alleviate these burdens. In Malhotra et al., participants receiving tirzepatide reported significant improvements in sleep-related impairment and sleep disturbance, as measured by the PROMIS questionnaires [1]. Similarly, the Blackman et al., noted that liraglutide led to significantly greater improvements in the “general health” and “activity level” domains of quality-of-life surveys [2]. By improving sleep quality and reducing daytime sleepiness, these treatments can be inferred to enhance cognitive functions, such as memory and attention, leading to improved performance at work and a reduced risk of accidents. This holistic improvement in both physiological health and patient-reported outcomes underscores the transformative potential of these therapies in the comprehensive management of OSA and obesity.

The use of GLP-1 RAs for treating OSA offers a promising alternative or adjunct to CPAP, particularly in patients with obesity or metabolic dysfunction. CPAP acts mechanically by maintaining an open airway during sleep, thereby reducing apneic events and improving oxygenation. However, its benefit is mainly dependent on adherence, which remains suboptimal in many patients due to discomfort and inconvenience, with adherence rates often falling below 50% in routine clinical practice [22]. GLP-1 RAs, in contrast, exert their effects systemically by reducing central adiposity, improving insulin sensitivity, and modulating autonomic tone and inflammation, key pathophysiologic drivers of OSA [1, 2]. These agents not only improve AHI and patient-reported sleep quality but also significantly impact weight loss and metabolic health, thereby targeting the underlying contributors to OSA progression. As a result, patients experience improved functional capacity and quality of life with a more tolerable treatment modality [23].

Furthermore, GLP-1 RAs have demonstrated significant reductions in major adverse cardiovascular events (MACE), heart failure hospitalizations, and all-cause mortality in patients with T2DM, and obesity, benefits not consistently observed with CPAP in randomized cardiovascular outcome trials [25–27]. Although GLP-1 RAs are currently more expensive than CPAP, their broad systemic benefits across multiple comorbidities (diabetes, obesity, cardiovascular disease) may enhance their cost-effectiveness, especially in high-risk populations [28]. CPAP, while cost-effective in adherent individuals, has limited impact on hard cardiovascular outcomes and no influence on metabolic risk. Therefore, GLP-1 RAs may offer a more comprehensive approach to OSA management, especially in populations where cardiometabolic risk reduction is a priority [27].

### Limitations

There are significant limitations to consider. First, heterogeneity in the study designs and populations could compromise the validity of pooled estimates. For example, Malhotra et al., included two parallel trials (with and without background positive air pressure (PAP) therapy). In contrast, Blackman et al., excluded participants who could use PAP, and Jiang et al. required all participants to use CPAP. This highlights differences in baseline risk and potential effect modification by concurrent therapy [1, 2, 7]. Furthermore, the inclusion of T2DM in Jiang et al. introduces a metabolic confounder that is not present in the other trials, which may affect both the response to GLP-1 agonists and the severity of OSA at baseline and follow-up [7].

Differences in intervention dosing and duration also present challenges. Malhotra et al. used tirzepatide at up to 15 mg weekly for 52 weeks, while Blackman et al. used liraglutide 3.0 mg daily for 32 weeks, and Jiang et al. used liraglutide up to 1.8 mg daily for 3 months. These variations not only impact the magnitude and kinetics of response but also limit the interpretability of any pooled effect as directly comparable across drugs and regimens [1, 2, 7].

Another limitation is the diversity in background treatments and co-interventions. All studies incorporated lifestyle counseling, but the intensity and structure of these interventions may have differed. Importantly, the Jiang et al. trial allowed for changes in background T2DM and hypertension medications, which could further confound outcomes [7].

Finally, the relatively short duration of follow-up in Blackman et al. (32 weeks) and Jiang et al. (3 months) limits the ability to assess the long-term durability and safety of the effect, compared to the 52-week follow-up in Malhotra et al. This restricts the potential to draw robust conclusions about the sustained impact of GLP-1 agonists on OSA and related outcomes [1, 2, 7].

Despite being the most prescribed GLP-1 receptor agonist worldwide [29], semaglutide has not been evaluated in any RCTs specifically targeting obstructive sleep apnea. While a recent scoping review of GLP-1RAs in OSA highlighted potential benefits, it noted that existing evidence largely stems from broader GLP-1 RA classes rather than semaglutide alone.

While GLP-1 Receptor Agonists (GLP-1 RAs) demonstrate substantial benefits through weight loss and cardiometabolic improvements, their maximal effect may be attenuated in certain patient subsets where non-obesity-related factors like genetic factors influencing respiratory drive, predominantly contribute to OSA. Such genetic predispositions could indeed limit the effectiveness of GLP-1 RAs like tirzepatide and semaglutide in patients whose OSA is primarily driven by these underlying physiological mechanisms rather than solely by excess adiposity. This provides a more balanced and critical view on the potential limitations of GLP-1 RA therapy, despite its significant cost, for patients with diverse OSA etiologies [30].

In summary, while a meta-analysis of these trials could yield valuable insights into the class effect of GLP-1 receptor agonists on OSA severity in populations with obesity (with or without T2DM), caution must be exercised in interpreting pooled results due to heterogeneity in population, intervention, and background therapy. Addressing these limitations would require careful subgroup analysis and sensitivity testing to ensure any conclusions are both statistically and clinically meaningful [1, 2, 7].

### Comparison of previous meta-analyses

Compared to prior studies, this meta-analysis offers several significant advancements in both methodological rigor and clinical insight into the effects of GLP-1 RAs on OSA.

Unlike previous meta-analyses that incorporated non-randomized studies, unpublished trials, or a mixture of study types, our analysis strictly included only RCTs, the highest level of clinical evidence. This contrasts with Li et al. [31], who included a prospective cohort study and a non-randomized controlled trial, and Yang et al. [32], who also combined RCTs with non-RCTs. By focusing exclusively on RCTs, our findings offer greater internal validity and minimize confounding and selection bias.

Our study is the first to perform detailed subgroup analyses evaluating the impact of CPAP use and specific drug class (tirzepatide vs. liraglutide) on outcomes such as AHI, systolic and diastolic blood pressure. These subgroup analyses uncovered a significantly greater AHI reduction with tirzepatide (MD: − 23.80 events/hour) compared to liraglutide (MD: − 5.20 events/hour; *p* < 0.0001), a difference that was not statistically dissected in earlier analyses. While Li et al. and Yang et al. [31, 32] also performed some subgroup analyses, ours are more clinically nuanced and statistically robust.

This meta-analysis encompasses the most recent and high-impact clinical trials, particularly those by Malhotra et al. [29], which investigated the role of tirzepatide in OSA. Although Li et al. [31] and Yang et al. [32] also include this trial, our analysis updates the pooled data and offers a more current interpretation, extending up to June 25, 2025.

While Kow et al. [33] and other predecessors primarily emphasized changes in AHI or weight, our work integrates cardiometabolic outcomes, as body weight, systolic blood pressure, and diastolic blood pressure, providing a holistic view of GLP-1 RA effects in OSA management. This aligns better with clinical needs, especially given the shared pathophysiology between OSA and cardiovascular/metabolic disorders.

Through leave-one-out sensitivity testing, we confirmed that our findings were stable and not driven by any single study. While some previous studies reported I² values or mentioned heterogeneity, they lacked robust sensitivity testing across all outcomes. This further enhances the credibility of our conclusions.

## Conclusion

This meta-analysis demonstrates that GLP-1 RAs significantly improve key clinical outcomes in patients with OSA. The therapy resulted in a significant reduction in apnea-hypopnea index, substantial weight loss, and lower blood pressure, effects that were consistent across subgroups and robust to sensitivity analyses. These findings support the use of GLP-1 RAs as a promising adjunctive strategy in OSA management, particularly in individuals with obesity and cardiometabolic risk, offering a pathway to address both respiratory and systemic complications of the disease.

## Data Availability

Not applicable.

## Abbreviations

AHI: Apnea-hypopnea index
BMI: Body Mass Index
CI: Confidence interval
CPAP: Continuous Positive Airway Pressure
DBP: Diastolic blood pressure
DPP-4: Dipeptidyl Peptidase-4
GIP: Gastric inhibitory polypeptide
GLP-1: Glucagon-like peptide-1
GLP-1 RAs: Glucagon-like peptide-1 receptor agonists
HbA1c: Glycosylated Hemoglobin
hsCRP: High-sensitivity C-Reactive Protein
MD: Mean difference
OSA: Obstructive Sleep Apnea
RCTs: Randomized controlled trials
SBP: Systolic blood pressure
T2DM: Type 2 Diabetes Mellitus

## Data availability

The data used in this meta-analysis were extracted from publicly available sources, including published randomized controlled trials. All relevant data supporting the findings of this study are included within the manuscript and supplementary materials. Due to the nature of this analysis, no new primary data were generated. Additional details can be made available upon reasonable request to the corresponding author.
